# Directed Evolution of the Transcriptional Regulator DntR: Isolation of Mutants with Improved DNT-Response

**DOI:** 10.1371/journal.pone.0029994

**Published:** 2012-01-19

**Authors:** Rosa Lönneborg, Edina Varga, Peter Brzezinski

**Affiliations:** Department of Biochemistry and Biophysics, Arrhenius Laboratories for Natural Sciences, Stockholm University, Stockholm, Sweden; Emory University School of Medicine, United States of America

## Abstract

The transcriptional regulator DntR, which previously has been isolated from bacterial strains capable of degrading 2,4-dinitrotoluene (DNT), was engineered in order to improve the ability to detect DNT. A directed evolution strategy was employed, where sequence diversity first was created by random mutagenesis in three subsequent rounds, followed by recombination of previously selected mutants. A *gfp* gene was used as a reporter for transcriptional activity mediated by DntR and cells with higher GFP expression after addition of DNT were sorted out using fluorescence-activated cell sorting (FACS). A DntR mutant, which displayed 10 times higher induction levels than wild-type DntR in response to DNT was isolated. This mutant still maintained low levels of *gfp* expression in the absence of DNT. The detection limit was ∼10 µM, a 25-fold improvement compared to wild-type DntR. The functional role of some substitutions found in this clone is discussed in the framework of the structural changes observed when comparing the recently determined structures of DntR with and without bound inducer ligand.

## Introduction

2,4-dinitrotoluene (DNT) is the main component in the vapor-phase derived from the explosive TNT [1]. The compound is one of the major contaminants found near TNT-producing facilities. Because it is toxic and presumably a human carcinogen there is an increasing need for sensitive detection of DNT. Several attempts have been made to develop biosensors for the detection of DNT, both *in vivo* systems with whole-cell biosensors [2], [3] and *in vitro* systems using purified proteins/peptides [4], [5]. The major challenge in developing a sensor for detection of DNT is to achieve both high sensitivity and high selectivity. Biological systems that have evolved over million of years can attain these properties, which has been taken advantage of in development of whole-cell biosensors for naturally occurring compounds such as mercury [6] and arsenite/arsenate [7], with detection limits reaching nanomolar levels. Biosensors for detection of xenobiotic man-made compounds pose a much greater challenge because the time-span to evolve the ability for binding and degradation of such compounds has been significantly shorter. The previously reported whole-cell biosensors for detecting DNT displayed detection limits in the range of 25 µM [3] to 125 µM [2], with a limited specificity [2].

Only very few nitro-aromatic compounds existed in Nature before the era of synthetic chemistry. Compounds such as nitrotoluenes and nitrobenzenes are man-made and used in the manufacturing of dyes, polymers, pigments and explosives. These compounds are often highly toxic and persistent because few pathways for complete degradation exist. Isolation of several species of bacteria and fungi capable of degrading nitro-aromatic compounds have been reported [8], [9]. Investigation of the degradation pathways in several organisms has revealed evidence of recent and ongoing evolution. Remnants of genetic material from horizontal gene transfer are often seen, and there is usually a scattered organization of the genes encoding the degrading enzymes, which are still poorly adapted to the substrate in question. In addition, there is usually primitive and inefficient regulation of the enzyme synthesis [10].

DntR is an example of such an inefficient regulator. It is a LysR-type transcriptional regulator (LTTR) that was found in two different *Burkholderia* strains capable of degrading DNT [11], [12]. DntR regulates the oxidative degradation pathway of DNT, but is poorly adapted to this task and DNT is a poor inducer for DntR-mediated transcriptional activity [13]. Instead, salicylate was found to act as a more efficient inducer. Although there is no functional role for salicylate in the DNT-degrading strains, it is an intermediary metabolite in the naphthalene-degrading pathways controlled by NahR [14] and NagR [15] and remnants of genes needed for naphthalene degradation were also found in the DNT degrading strains. NahR has 61% amino-acid sequence homology to DntR while NagR differs from DntR only by two amino-acid residues (T46A and N49K), clearly indicating recent divergence.

More recently NtdR/NbzR, another homologue to DntR was found in two different bacterial strains capable of degrading 2-nitrotoluene and nitrobenzene respectively [16]. NtdR was reported to react on DNT and several other nitro-aromatic compounds that were unable to function as inducers for DntR [13], [16], [17], although only seven amino acid residues differ between the two proteins.

**Figure 1:**
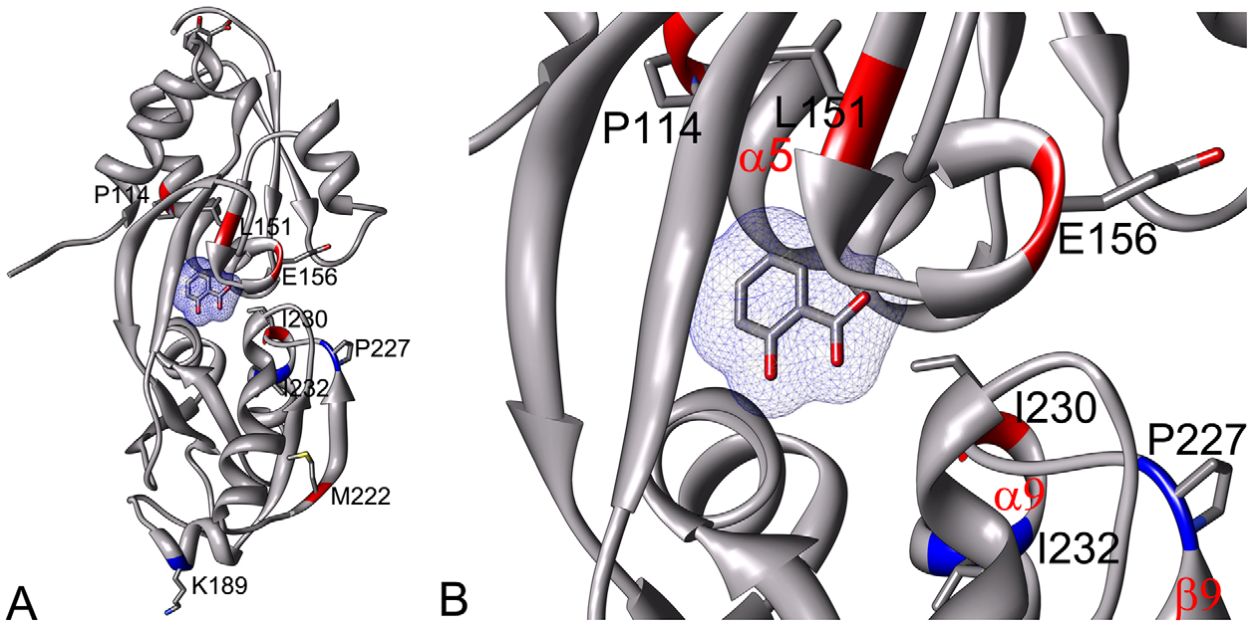
Structure of DntR. (A) Left: The ribbon structure of one inducer-binding domain of DntR with salicylate (salicylate is co-crystallized with the ΔN90-DntR construct in the fully closed state, PDB ID 2Y7P). The enriched mutations from the first four generations of libraries are shown colour-coded from red (occurring>10 times) to green (occurring 3–4 times). Right: Table listing the enriched mutations with the same colour-coding as in the picture above. The number of occurrences is calculated from all mutations occurring in randomly selected clones (a total of 82 clones) from library 1 to library 4. (B) Ribbon representation of the IBC of DntR with salicylate. The side-chains are shown for the residues where the enriched mutations are found. Also marked is the position of helix α5, helix α9 and β-strand β9 and the side-chain of D105.

DntR is functionally active as a homo-tetramer, where each monomer could be divided into an N-terminal DNA-binding domain (DBD) with a winged helix-turn-helix motif connected to a C-terminal inducer-binding domain (IBD) (**Figure 1**) via a linker region. The IBD could be further divided into two subdomains (RD1 and RD2) connected with a hinge region, with the inducer binding cavity (IBC) situated between RD1 and RD2. Structures of the full-length DntR have been determined with acetate or thiocyanate in the IBC [18]. Inducer binding is suggested to cause changes of the global protein conformation where re-positioning of the DBDs would relax the angle of bound promoter-DNA and thereby trigger transcriptional activation [13], [18], [19]. Very recently, we obtained structural data of a truncated DntR protein without the DBD-domain, with and without the inducer salicylate [20]. These data reveal two main conformations of the IBD, where salicylate binding induces a rotation of RD2 relative to RD1 such that several loops in RD2 move closer to loops in RD1, which results in wrapping of the salicylate between RD1 and RD2.

To this date, most efforts of altering the properties of transcription factors have relied on a rational-design strategy. However, it is commonly difficult to predict the effect of a single mutation at a functional level, especially when ligand binding is coupled to global structural changes [21]. A few studies have instead been based on the use of directed evolution to alter the properties of transcription factors [2], [22], [23]. In the present study we used the “NtdR” variant of DntR as a starting point and applied a directed evolution strategy to change the inducer specificity of the transcription factor DntR towards improved DNT response. A clone with a satisfactory repressor ability and a dramatic improvement of both the level of response and sensitivity for DNT was isolated. The effect of some substitutions found in this clone are also discussed in the framework of observed differences in the structures of DntR with and without the inducer salicylate [20].

## Results and Discussion

### Library construction, selection and screening

An “NtdR” mutant of DntR that showed broader inducer specificity than wild-type (wt) DntR (**Table S1**) was selected as a starting template in the search for DntR variants with improved response to DNT. The “NtdR” mutant was chosen due to this broadened inducer response, since a protein with a broad specificity is more likely to evolve its specificity towards a new ligand than a protein with a narrow specificity [2], [24], [25], [26]. A *gfp* gene [27] was used as a reporter for DntR-mediated transcriptional activation, and cells displaying an increase in GFP fluorescence after exposure to DNT were sorted out from the constructed libraries using FACS.

**Figure 2:**
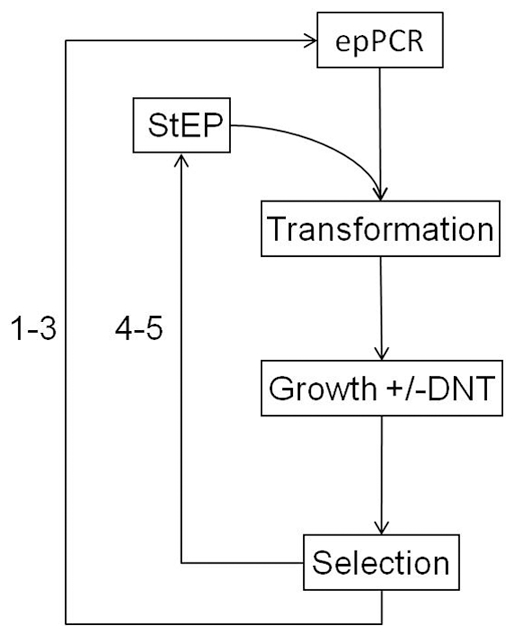
Overview of the directed evolution process. Five libraries were created in subsequent order, as indicated by the numbers 1–5. Library 1–3 were created using epPCR with Mutazyme II polymerase. The library size was in the range of 1–7×10^7^ transformants/library. Library 4 was created using a modified variant of StEP using Mutazyme II polymerase, resulting in recombination of clones selected from library 3 with additional point mutations introduced by Mutazyme II. The best responding clones selected from library 4 were then recombined with StEP, this time using Pfu Polymerase. The success of recombination was confirmed by sequencing of 8 clones from each of library 4 and 5. Library 1–4 was grown in LB, while Library 5 was grown in both LB and M9*, with selection performed on cells grown in M9*. Each library was sorted 2–4 times (sorting and regrowth prior to another library generation indicated by dashed arrow).

**Figure 3:**
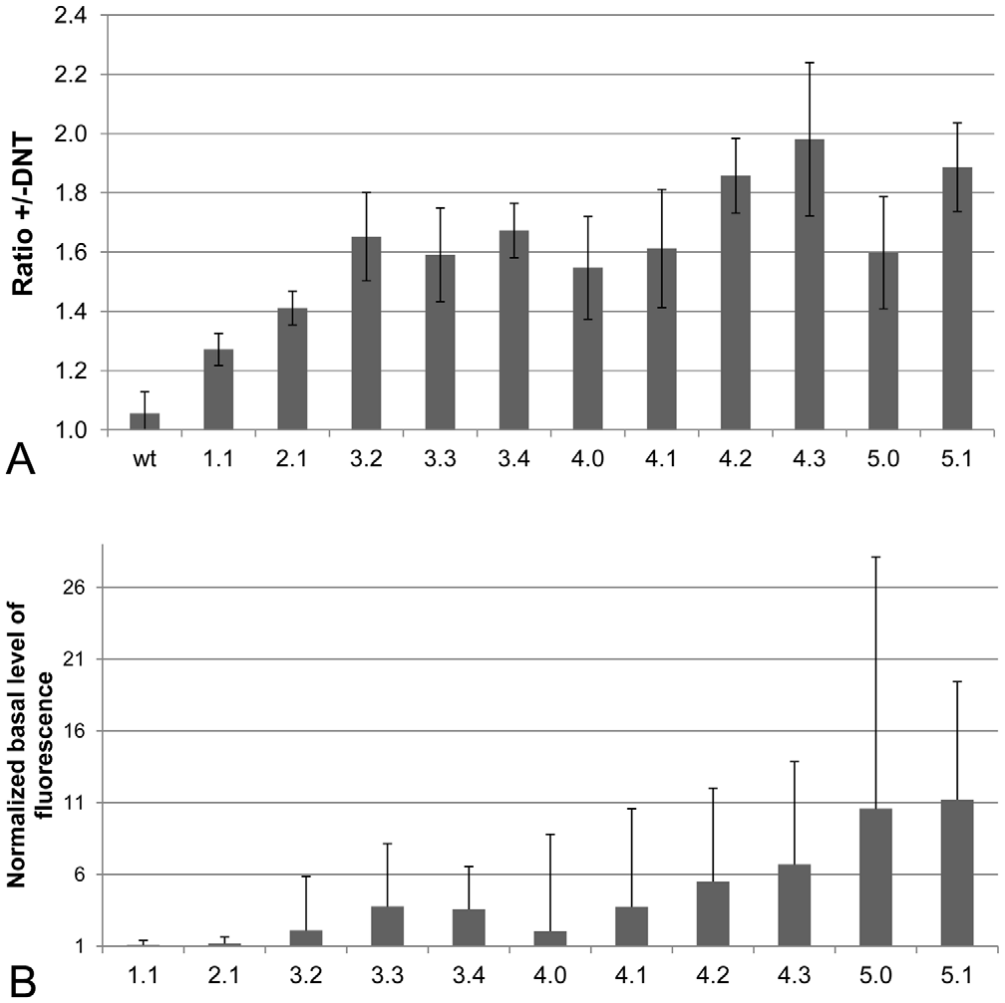
The response to DNT and the basal level of fluorescence. The diagram shows the mean response to DNT (A) and the mean basal level of fluorescence (B) for randomly selected clones from consecutive steps during the sorting procedure until the first sorting of library 5. The first number indicates the library, and the second number indicates the sorting step for that library. A) The response to DNT was measured for 5–10 clones for each sorting step. For each clone the response was calculated as the ratio between the mean fluorescence of 10000 cells grown in the presence of 500 µM DNT and the mean fluorescence of 10000 cells grown in the presence of the solvent DMSO. Shown in each bar is the mean for all the analyzed clones in that sorting step and in error bars are the standard deviation of the DNT response for these clones. As a comparison the mean response to DNT for wt DntR is shown with the standard deviation based on five independent data sets. B) The basal level of fluorescence is calculated as the ratio between the fluorescence of the population with DMSO added compared to the fluorescence of a WT DntR control with DMSO added for the same clones as in A). Clones were also analyzed for library 5, but since these were grown in M9* and first subjected to a screen on plate instead of randomly selected, there is no comparable data available.

The directed evolution process was performed in five consecutive rounds (see overview in **Figure 2**). The first three rounds employed mutagenic PCR with an average mutation frequency of ∼2 mutations/gene fragment. Randomization was applied to residues 38–301, excluding the first part of the DBD. In round 4 and 5, libraries were created by recombination, using a variant of staggered PCR (StEP). An average increase in the DNT-response could be seen when analyzing randomly picked clones from the subsequent sorts (**Figure 3A**). This was in general also accompanied by an increase in the basal level of fluorescence (**Figure 3B**). To reduce the basal level of fluorescence, a switch of growth media was performed in round 5 from LB to M9*.

By switching from the rich LB medium to M9*, a subpopulation with lower basal level of fluorescence in M9* than in LB was observed. Upon addition of DNT, we observed a shift to higher fluorescence for this subpopulation. Consequently, this subpopulation was sorted out, thereby removing the constitutively active clones with high basal activities in both growth media and enriching the clones with lower basal level of fluorescence in M9*. Two additional rounds of sorting was performed, and from these sorts 132 clones were screened individually, and 11 unique clones with a DNT-response ranging from 3–12 fold induction with 500 µM DNT were identified (**Table 1**).

**Table 1:** The sequences (compared to the sequence of wt DntR) of the original templates and the clones with >3-fold induction in response to DNT when grown in M9 listed together with the response to 500 µM DNT and 500 µM salicylate expressed as the fold of induction.

Protein variant	SAL	DNT	Sequence
wt	10.4±0.9	1.2±0.1	
NtdR	12.6±0.6	1.9±0.2	**T46A, N49K, I74V, H169L, K189R, P227S, I232V**
**5.3P3c19**	**12.0±0.9**	**11.6±1.3**	S40A, **T46A**, **N49K**, L54V, **I74V**, P114Q, L151F, E156K, **K189R**, M222I, **P227S**, I230N, **I232V**
5.3P3c21	12.7±0.9	4.8±0.4	**T46A, N49K**, L54V, P114Q, M222I, **P227S**, I230N, **I232V**
5.2R3c24	10.1±0.5	4.1±0.3	S40A, **T46A, N49K**, L54V, **I74V**, N203Y, M222I, **P227S, I232V**
5.2R3c5	8.9±0.5	4.1±0.4	S40A, **T46A, N49K, I74V**, R165H, **K189R, P227S**, I230N, **I232V**
5.2R3c18	8.5±0.6	4.0±0.2	S40A, **T46A, N49K, I74V**, R165H, **P227S**, I230N, **I232V**
5.2R3c13	6.9±0.4	3.8±0.3	**T46A, N49K**, L54V, **I74V**, T96R, N203Y, **P227S, I232V**, K280R
5.3P3c27	6.7±0.6	3.7±0.2	**T46A, N49K**, L54V, P114Q, R165H, **K189R**, **P227S**, I230N, **I232V**
5.3P3c43	4.6±0.2	3.7±0.2	**T46A, N49K**, L54V, A70S, **I74V**, P114Q, R165H, **P227S**, I230N, **I232V**
5.2R3c11	7.4±0.3	3.5±0.3	**T46A, N49K**, L54V, A70S, **I74V**, T79A, T96R, R165H, **P227S, I232V**
4.2c1	5.4±0.1	3.6±0.4	**T46A, N49K**, L54V, **I74V**, **P227S**, I230N, **I232V**
5.3P2c31	4.4±0.7	3.2±0.1	S40A, **T46A, N49K**, L54V, A70S, **I74V**, R165H, N203Y, M222I, **P227S**, I230N, **I232V**

The mean fluorescence was measured for a cell population of 10 000 cells for each sample with the standard error based on at least three independent experiments shown in parenthesis. Clones are named after the library and sorting which they were isolated from followed by a unique clone label. The substitutions derived from the NtdR template are marked in bold.

### Clones with improved DNT-response

**Figure 4:**
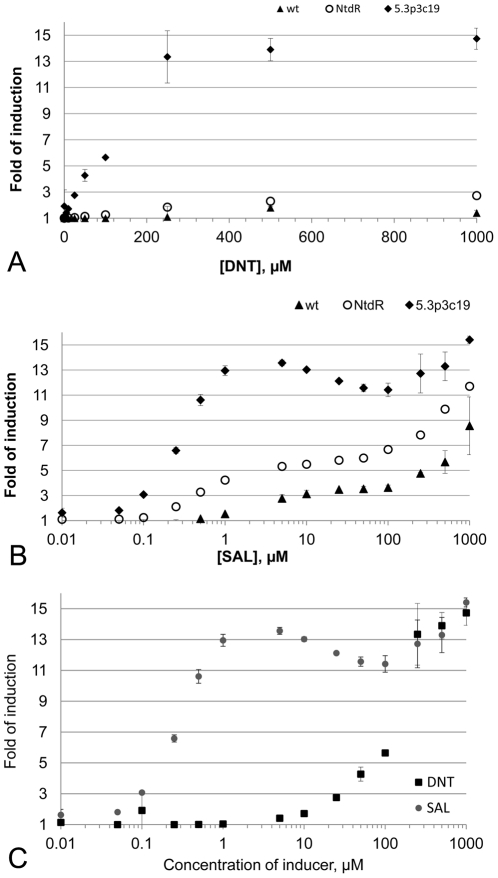
The response to DNT and salicylate. The measurements were done in the concentration range .10 nM-1000 µM for wt, NtdR and the 5.3p3c19 mutant grown in M9 medium, respectively (The water solubility limit of DNT is slightly above 1 mM). The response was measured as fold of induction and is calculated as the mean fluorescence of a cell population grown with the added concentration of inducer divided by the mean fluorescence of a cell population grown with addition of the solvent DMSO. Shown in error bars are the standard deviations. (A) The response to DNT measured as the fold of induction for wtDntR (filled triangles), NtdR (open circles) and 5.3p3c19 (filled diamonds). (B) The response to salicylate measured as the fold of induction for wtDntR (filled triangles), NtdR (open circles) and 5.3p3c19 (filled diamonds). (C) The response to DNT (black squares) and salicylate (grey circles) for the 5.3p3c19 clone (logarithmic scale).

Several clones with a 3–12 fold response to 500 µM DNT were isolated in the second and third sorting round of library 5 (**Table 1**). Also the 4.2c1 clone from the second sorting round of library 4 that was used as one of the templates for library 5, displayed a more than 3-fold induction with DNT in M9. Eight out of 11 of the best-responding clones in library 5 carried the I230N substitution derived from the 4.2c1 mutant, among them the best-responding clone 5.3p3c19. This clone showed an increase in fluorescence by a factor of ∼12 upon addition of 500 µM DNT, compared to wt DntR. This mutant also had an increased response to salicylate, 2-nitrobenzoate, 4-nitrobenzoate and benzoate compared to both wt DntR and NtdR (**Table 2**). The response in the range of 250–1000 µM of both salicylate and DNT were at similar levels (13–15 fold induction) for this mutant (**Figure 4A–C**). The level of response to 250–1000 µM DNT for the 5.3p3c19 mutant was higher than that of wt DntR to salicylate in the same concentration range. This mutant apparently displayed both a large improvement in DNT response and a general improvement of the sensitivity both for the original inducer salicylate and for all tested compounds having a carboxy-group. The 5.3p3c19 mutant shows a general improvement in sensitivity, with a detection limit of ∼10 µM for DNT and ∼50 nM for salicylate, as compared to ∼250 µM and ∼250 nM, respectively for NtdR and ∼250 µM and 1 µM respectively for wt DntR (**Figure 4C**).

**Table 2:** The response to a set of potential inducers for the original templates and the 5.3p3c19 clone grown in M9 22 h after induction.

Protein variant	SAL	DNT	2-NT	2-NB	4-NT	4-NB	BEN	Basal F
wt DntR	540±80	70±30	7±4	30±30	9±1	32±8	−1±2	0
”NtdR”	820±30	110±20	−15±15	40±30	0±27	260±70	150±60	60±35
5 5.3p3c19	1200±90	1200±130	21±16	820±120	65±35	530±70	1130±150	40±23

The response is expressed as the % increase in fluorescence when 500 µM of the potential inducer is added compared to the control where only solvent (DMSO) is added. The mean fluorescence was measured for a cell population of 10 000 cells for each sample with the standard error based on at least three independent experiments shown. The basal level of fluorescence (Basal F) is compared to that of WT DntR grown in M9 at the same signal amplification and is measured as % increase in fluorescence for mutant grown with DMSO/fluorescence for wt grown with DMSO. (Wt grown in LB gives a 100%(±30) increase in fluorescence compared to wt grown in M9). SAL: salicylate, DNT: 2,4-dinitrotoluene, 2-NT: 2-nitrotoluene, 2-NB:2-nitrobenzoate, 4-NT: 4-nitrotoluene, 4-NB: 4-nitrobenzoate, BEN: benzoate.

### Structural significance of some substitutions obtained during the selection process

Sequencing of clones from each step of the sorting and selection of the first four libraries yielded a total of 81 clones carrying 291 mutations that altered the amino-acid composition. It was apparent that changes in some residues were reoccurring in several clones and were enriched during the sorting process, whereas ∼45% of the unique point mutations occurred only once (∼80% once or twice) and appear to be evenly distributed over the randomized *dntR* fragment.

The most frequently mutated residue was H169, but substitutions at this site were not found among any of the clones with improved DNT-response (the role of this residue is discussed in [28]).

The enriched substitution Arg165His (**Figure 1**) is found at about the same location as Arg156 in BenM, another member of the LysR type family induced by benzoate. In BenM, an R156H mutant was reported to have higher basal level of transcriptional activity than wild-type BenM [29].

When comparing the recently published structures of the IBD with and without salicylate bound [20], it is seen that inducer binding results in rotation of RD2 towards RD1, resulting in large movement of the α9 helix that also becomes partly unstructured. Also, the β8-α8 and β9-α9 loops in RD2 close towards the β4-α6 loop in RD1 (see Figure 4 in Devesse *et al.* for details [20]). In the closed conformation, these loops enclose the salicylate bound in the IBC, expelling water from the RD1–RD2 interface. In the best-responding clone, 5.3P3c19, an I230N substitution is found. I230 is situated in the β9-α9 loop (also the enriched substitution H228N is found in this loop). The I230N substitution would presumably result in a steric clash with the opposing D105 in α5 of RD1 upon transition of the IBD to the closed conformation (ligand bound). The side chains of Y110, F111 and I106 in this α5 helix constitute a hydrophobic wall in the IBC (**Figure 1B**). Repositioning of α5 due to the interactions between N230 and D105 could increase the volume of the hydrophobic part of the IBC which is expected to favor DNT-binding.

The combination of I230N with L151F is suggested to be the main reason for the improved DNT-response of the best-responding 5.3P3c19 clone compared to the clones with only 3–4 fold induction that carries the I230N substitution but not L151F (**Table 1**). The carbonyl oxygen of Leu151 forms a water-mediated interaction with salicylate in the IBC [20], and substitution with a Thr was previously suggested in order to reduce salicylate binding and facilitate DNT-binding [18]. However, the L151T substitution resulted in a lowered response to salicylate, but no change of inducer specificity [13]. The enriched L151F substitution found in the best responding mutant 5.3p3c19 is expected to change the position of the carbonyl oxygen of this residue, suggested to increase the volume of the hydrophobic part of RD1 close to the IBC, because the side-chain of the introduced F151 would have to move in order not to clash with F111.

In conclusion, by using several rounds of random mutagenesis followed by recombination of clones with a modest DNT-response, a DntR variant with greatly improved DNT-response was isolated. This variant also displayed a higher sensitivity to several other aromatic compounds, including the original inducer salicylate. Interestingly, the sensitivity observed for salicylate in this study is in fact higher than in studies aiming at improving the sensitivity of salicylate biosensors [30], [31].

## Materials and Methods

### Chemical reagents and enzymes

Salicylic acid, 2-nitrotoluene, 2-nitrobenzoic acid, 2,4-dinitrotoluene and 2-hydroxytoluene (o-cresol) were all purchased from Sigma-Aldrich. Benzoic acid, 4-nitrobenzoic acid and 4-nitrotoluene were purchased from VWR International. All chemicals were of the highest grade available. For the flow cytometric analysis, 500 mM stock solutions of each compound were prepared in dimethylsulfoxide (DMSO). The stock solutions were kept frozen between usages. Restriction enzymes were purchased from Fermentas and T4 DNA ligase was purchased from New England Biolabs. For the random mutagenesis, the GeneMorph® II Random Mutagenesis kit (Stratagene, La Jolla) was used.

### Library construction, selection and screening

The introduction of mutations to create the starting template “NtdR” from wtDntR was done with the QuikChange Site-Directed-Mutagenesis kit (Stratagene, La Jolla). For an overview of the work process, see **Figure 2**.

For the first library, the primers GACATCGACTTGAATCTGCTGG and CATTGGGATATATCAACGGTGG were used together with MutazymeII polymerase (Stratagene, La Jolla), which amplifies an 1122 bp fragment containing the *dntR* gene from the pQE60DntR plasmid (construction of this plasmid has been described previously [13]). In each PCR tube, 90 ng of plasmid (corresponding to 45 ng of target DNA) carrying the “*ntdR”* gene was used. PCR was carried out using a program of 95°C for 5 min followed by 30 cycles of 95°C for 45 s, 55°C for 45 s and 72°C for 1 min 30 s. For the second and third library, the primers GACATCGACTTGAATCTGCTGG and GCCACCTGACGTCTAAGAAACC were used to amplify a 1061 bp fragment containing the *dntR* gene library variants from the pQE*dntR*:P_DNT_:*gfp* plasmid. PCR was performed as above, using 90 ng of plasmid in each tube.

For the fourth library, plasmids containing the clones from the third sorting of the third library were used as templates in a combination of staggered extension process PCR and epPCR by using the mutazyme and the following PCR programs: I) 95°C 7 min followed by 30 cycles of 95°C for 30 s and 60°C for 5 s. II) 95°C for 5 min followed by 40 cycles of 95°C for 45 s, 55°C for 45 s and 72°C for 1 min 30 s. For creation of the fifth library the same PCR programs were used with six selected clones from the fourth library as templates in an equimolar mixture, but this time Pfu polymerase was used in the process in order to achieve recombination without additional introduction of point mutations.

For all libraries the PCR products were purified from gel with the E.Z.N.A Gel Extraction Kit (Omega Bio-tek). The PCR products were digested with EagI and BglII. Digestion of the fragment resulted in a 775 bp fragment, corresponding to the *dntR* gene with the first 97 nucleotides excluded (corresponding to the 32 first N-terminal amino-acids). This fragment was purified from the gel, and then ligated into the EagI and BglII digested pQE*wtdntR*:P_DNT_:*gfp* plasmid. Following ethanol precipitation, the ligated library (a total of 5–10 µg DNA) was transformed into E. *coli* DH5α cells through electroporation. The total number of transformants was estimated from plating a dilution series to be in the range of 1–7×10^7^ transformants per library with a background of religated/undigested pQE*dntR*:P_DNT_:*gfp* plasmid ranging from 3–30%.

For each library and each sorting of the library, 5–10 clones were sequenced to estimate the mutation frequency. For the first library the mutation frequency was estimated to 2.2 mutations/amplified gene fragment, based on sequencing of 10 clones. For the next libraries, more recombinations with the wt DntR sequence were observed, making an estimation of the mutation frequency difficult.

Since the fourth library was constructed from a pool of mutants derived from the third library using the MutazymeII polymerase that also introduces additional point mutations, the number of recombinations could not be estimated in this library. In the fifth library that was constructed using Pfu polymerase and where all of the sequences selected for recombination were known, sequencing of 8 clones in the unsorted library gave an average minimum number of recombinations of 3 per fragment, where fragments from all starting templates were represented, suggesting that the simplified (StEP) protocol used had been successful in achieving good recombination.

### Growth conditions

The first four libraries were grown in Luria-Bertani (LB) medium, while sort 1 to 3 of library 5 was grown in parallel in LB and a modified M9 medium, M9* (1×Difco M9 minimal salts, 1 mM MgSO_4_, 0.1 mM CaCl_2_, 0.2% glucose and 1%LB). For later analysis of DNT-responding clones, M9 (1×Difco M9 minimal salts, 1 mM MgSO_4_, 0.1 mM CaCl_2_, 3 µM thiamine, 0.2% glucose and 0.7 mg/ml of each amino acid) was used. With all growth medias, the cells were grown at 30°C with shaking and supplied with 100 µg/ml ampecillin for maintenance of the pQE*dntR*:P_DNT_:*gfp* plasmid. The transformed colonies containing the ligated library of *dntR* variants on the pQE*dntR*:P_DNT_:*gfp* plasmid were pooled in 100 ml growth medium, grown for 2.5 h and pelleted. Freeze cultures were prepared from half of the culture, while the other half was used for plasmid preparation. Prior to analysis and sorting, a freeze culture of the library was grown in 25 ml of growth medium for 2 h. This culture was used to inoculate 2×100 ml culture so that the OD_600_ at inoculation was 0.05. 2 h later one culture was induced with 500 µM DNT and DMSO was added to the other. The cultures were grown for 4 h in the presence or absence of DNT. The cells were pelletted down, and diluted in PBS prior to flow cytometric analysis and sorting. After sorting, cells were plated and regrown using the same protocol as above.

Individual clones from the fifth library were plated on M9*+agarose plates supplied with or without 500 µM DNT. Clones that appeared more fluorescent on the plate supplied with DNT were grown in liquid media as below.

Individual clones from all steps were grown overnight in 5 ml LB medium, and then inoculated to 1 ml growth media with a start OD_600_ of 0.05. (The rest of the overnight cultures were used for plasmidpreparation followed by sequencing). After 2 h, 500 µM DNT was added, and 4 h/22 h later cells were taken out for FACS analysis. Selected clones were in addition also grown in the presence of 500 µM of a set of compounds with similarity to salicylate and/or DNT (2-nitrotoluene, 2-nitrobenzoate, 4-nitrotoluene, 4-nitrobenzoate and benzoate) with the same growth conditions as above.

The 5.2p3c19 clone, wt DntR and “NtdR” was also grown in M9 with concentrations of salicylate and DNT in the range of 10 nM-1000 µM, which were added from 1000× stock solutions for all concentrations to cell cultures grown as above.

### Flow cytometric analysis, sorting and screening

The flow cytometric analyses and sorting of libraries were performed on a FACS Vantage SE stream-in-air flow cytometry instrument (BD Biosciences, San Jose, CA, USA). The water-cooled argon ion laser was aligned prior to each analysis using flow cytometry beads for 488 nm excitation (Life technologies, Paisley, UK), and the same instrument settings were used for each time of analysis. Fluorescence was detected via a 530±15 nm (green) band pass filter. For each cell sample, data from 10 000 events were collected. For analysis of single clones, a FACS Calibur instrument (BD Biosciences, San Jose, CA, USA) was used with the same settings each time.

The cell population of the DNT-induced library was compared with the cell population of the non-induced library and with a control culture expressing wt DntR. A first gate was set to exclude cells with too high side and forward scatter. For the first four libraries, a gate was set to sort out cells with high fluorescence in the DNT-induced population while minimizing overlap with the population of the non-induced library. For the first three generations, 4–5% of the most fluorescent cells were sorted out in each sorting, with an overlap between the induced and non-induced population ranging from 15%–43%. In general, the overlap increased for each round of sorting due to enrichment of clones giving increased basal level of fluorescence. After regrowth of the sorted cells, the cells were sorted for 1–3 more rounds. For the fourth library where there was a prominent tail with higher fluorescence, 6–20% of the cells were sorted out with an overlap of 32–57%. In the first two rounds of sorting of the fifth library, cells with intermediary fluorescence (higher than the WT control+DNT, but lower than a large part of the population with mainly constitutively active mutants) were sorted out in the first round. In the following round, clones with higher fluorescence in response to DNT were again sorted out.

One particular challenge when selecting for DntR variants with increased response to DNT using GFP fluorescence as a sorting parameter, was the accumulation of clones with higher basal level of fluorescence in the absence of any added inducer. These clones were easily enriched after several rounds of cell sorting using high fluorescence as the major sorting parameter (**Figure 3B**). To reduce the number of false positives (i.e. inducer-independent phenotypes), another round of sorting was only done if reanalysis of the sorted cells grown with and without DNT showed that the ratio +/−DNT was increased compared to the previous round of sorting. A negative selection step where clones with low fluorescence in the absence of DNT were sorted from the second sort of the third library did lower the basal level of fluorescence in the sorted population, but the ratio +/−DNT was also reduced at this step. Later selection of clones in library 5 with lower fluorescence in M9 led to an increase in the ratio +/−DNT.

## Supporting Information

Table S1 **The response to a set of potential inducers for wt, NtdR and the 5.3p3c19 clone grown in LB.** The response is measured in the same way as in table 1. Data is based on at least three independent experiments with the standard error shown. The basal level of fluorescence is compared to that of WT DntR grown in LB at the same signal amplification.
(PDF)
