# Supplementary material for: Directed Evolution of the Transcriptional Regulator DntR: Isolation of Mutants with Improved DNT-Response
Source: PLoS One. 2012 Jan 19;7(1):e29994. doi: 10.1371/journal.pone.0029994 (PMC3261848; doi:10.1371/journal.pone.0029994)
Supplement: Table S1 — The response to a set of potential inducers for wt, NtdR and the 5.3p3c19 clone grown in LB. The response is measured in the same way as in table 1. Data is based on at least three independent experiments with the standard error shown. The basal level of fluorescence is compared to that of WT DntR grown in LB at the same signal amplification. (PDF) [file pone.0029994.s001.pdf]

Protein variant	SAL	DNT	2-NT	2-NB	4-NT	4-NB	BEN	Basal F*
wt	1222±336	23±3	5±2	3±3	9±3	44±12	17±4	0**
NtdR	1278±289	15±11	3±6	29±11	4±15	367±31	130±13	10±2
5.3p3c19	675±11	262±32	6±5	184±23	17±12	150±14	470±19	29±7

The response to a set of potential inducers for wt, NtdR and the 5.3p3c19 clone grown in LB. The response is measured in the same way as in table 1. Data is based on at least three independent experiments with the standarderror shown. The basal level of fluorescence is compared to that of WT DntR grown in LB at the same signal amplification.
*% increase in fluorescence for mutant with DMSO only compared to fluorescence for wt DMSO only in same growth conditions
**The basal level of fluorescence for wt in each growth medium is set as a reference for the other variants in the same growth medium. Wt grown in LB gives a 104%(±27) increase in fluorescence compared to wt grown in M9*

Supplementary table 1
